# Comparable short‐term anterior knee laxity and extension outcomes following anterior cruciate ligament reconstruction using TightRope II RT, TightRope RT and EndoButton CL: A retrospective matched cohort study

**DOI:** 10.1002/jeo2.70506

**Published:** 2025-11-14

**Authors:** Dzan Rizvanovic, Christoffer von Essen, Riccardo Cristiani, Anders Stålman

**Affiliations:** ^1^ Department of Molecular Medicine and Surgery, Stockholm Sports Trauma Research Center Karolinska Institutet Stockholm Sweden; ^2^ Capio Artro Clinic, FIFA Medical Centre of Excellence Sophiahemmet Hospital Stockholm Sweden

**Keywords:** ACL, ACLR, adjustable loop, cortical suspensory device, femoral fixation, fixed loop, hamstring

## Abstract

**Purpose:**

To compare short‐term outcomes in anterior knee laxity and extension deficit 6 months after primary anterior cruciate ligament (ACL) reconstruction (ACLR) with TightRope II RT, TightRope RT or EndoButton CL for femoral fixation.

**Methods:**

A retrospective matched cohort study was conducted, including 864 patients (288 per group) who underwent primary ACLR with hamstring tendon autografts at Capio Artro Clinic, Stockholm, Sweden, between 2005 and 2024. Patients were matched 1:1:1 based on age, sex, concomitant medial or lateral meniscal resection, cartilage injury, and time from injury to surgery. Anterior knee laxity was assessed preoperatively and at 6 months postoperatively using the KT‐1000 arthrometer. Extension deficit was defined as >5° from anatomical zero and measured at 6 months using a goniometer.

**Results:**

Preoperative mean side‐to‐side (STS) laxity was 3.8  ± 2.4 mm for TightRope II RT, 3.6  ± 2.4 mm for TightRope RT, and 3.8  ± 3.0 mm for EndoButton CL. At 6 months, STS values were 1.7  ± 1.6 mm (TightRope II RT), 1.8  ± 2.5 mm (TightRope RT), and 2.0  ± 2.3 mm (EndoButton CL). No significant differences were observed between groups at either time point. STS > 5 mm occurred in 1.7% (5/287) of TightRope II RT patients, 5.6% (8/142) with TightRope RT, and 5.8% (15/258) with EndoButton CL, with no significant differences. Extension deficit was observed in 0.4% (1/244), 1.5% (3/203), and 0.8% (2/259), respectively, without significant differences.

**Conclusion:**

TightRope II RT demonstrated comparable short‐term outcomes in anterior knee laxity and extension to both TightRope RT and EndoButton CL. These findings support the use of TightRope II RT as a reliable femoral fixation method in primary ACLR.

**Level of Evidence:**

Level III, retrospective comparative study.

**Abbreviations**
ACL: anterior cruciate ligament
ACLR: anterior cruciate ligament reconstruction
EB: EndoButton CL
IQR: interquartile range
n.s.: not significant
SD: standard deviation
STS: side‐to‐side
TR: TightRope RT
TRII: TightRope II RT

## INTRODUCTION

Secure femoral fixation is pivotal to the success of hamstring‑tendon anterior cruciate ligament (ACL) reconstruction (ACLR), ensuring graft stability during the phases of tendon‑to‑bone healing [17]. Cortical suspensory devices have become the predominant method for femoral fixation in ACLR [23]. These devices are available with either a fixed loop, which has a predetermined length, or an adjustable loop that allows intraoperative shortening and retensioning [15].

Although fixed loop devices have been shown to potentially offer better biomechanical properties than adjustable loop devices, these differences decrease when the latter are retensioned after tibial fixation [18]. Prior research has shown no differences in revision rates, knee laxity or subjective knee function between fixed and adjustable loop devices [8, 9]. Most comparative studies to date have focused on the first‑generation TightRope RT (Arthrex), an adjustable‐loop device, and the fixed‐loop EndoButton CL (Smith & Nephew) [8]. In 2021, a second‐generation device, TightRope II RT (Arthrex), was introduced with design modifications aimed at improving graft tensioning and mitigating cyclic displacement. Clinical data on this implant are limited, and studies are needed to clarify its performance relative to both its predecessor and fixed‐loop devices.

When evaluating femoral fixation devices, both anterior knee laxity and postoperative extension deficit are important clinical outcomes. Increased side‐to‐side (STS) laxity greater than 5 mm at 6 months after primary ACLR has been associated with a higher risk of revision surgery [5]. Additionally, excessive graft tensioning may contribute to postoperative extension loss, which may delay rehabilitation and impair knee function [21].

The purpose of this study was to compare short‐term outcomes regarding STS laxity and extension deficit 6 months after primary ACLR performed with TightRope II RT, TightRope RT or EndoButton CL femoral fixation. It was hypothesized that the TightRope II RT would yield outcomes equivalent to those observed with the first‐generation TightRope RT and the EndoButton CL.

## METHODS

Ethical approval was obtained from the Swedish Ethical Review Authority (2024‐07166‐02).

### Study population

Patient data were retrieved from our institutional clinic database. Individuals who had undergone primary ACLR between 2005 and 2024 were screened for eligibility. Inclusion was limited to patients who received a hamstring tendon autograft with a femoral cortical suspensory device (TightRope II RT, TightRope RT or EndoButton CL) and an AO bicortical screw as a post for the tibia. Additionally, included patients had no associated ligament injuries requiring surgical treatment, and they had not undergone any previous contralateral ACLR or revision procedures.

Patients who received TightRope II RT fixation formed the index group and were matched in a 1:1:1 ratio with those treated using TightRope RT and EndoButton CL devices. The number of participants was determined by the number of patients available in the TightRope II RT group. Matching was performed based on age (±2 years), sex, medial meniscus resection (yes/no), lateral meniscus resection (yes/no), presence of cartilage injury (yes/no), and time from injury to surgery (<3, 3–6, 6–12, 12–24, >24 months).

Capio Artro Clinic, Stockholm, Sweden, is a high‐volume center with over 700 primary ACL reconstructions and revisions performed annually during the study period [20]. All ACLRs were performed by fellowship‐trained sports medicine surgeons.

### Surgical technique and rehabilitation

All patients underwent single‐bundle ACLR using a quadrupled semitendinosus tendon. If the graft was deemed insufficient in length or diameter (<8 mm), the gracilis tendon was additionally harvested. The minimum graft length was 60 mm, with at least 15 mm in each bone socket. Femoral tunnel drilling was performed using the anteromedial portal technique. Femoral fixation was achieved with a TightRope II RT, TightRope RT, or EndoButton CL device, according to the respective manufacturers' instructions. During the study period, device selection was based on in‐clinic availability. After graft cycling, tibial fixation was performed by securing Ethibond no. 2 sutures (Ethicon) over an AO bicortical screw with a washer (Smith & Nephew), used as a post. Intraoperative laxity was confirmed using the Lachman test.

Meniscal repairs were carried out using an all‐inside arthroscopic technique with either Fast‐Fix (Smith & Nephew) or FiberStitch (Arthrex) devices for tears located in the posterior and middle segments of the meniscus. Tears located in the anterior region were repaired using an outside‐in technique with No. 0 PDS sutures (Ethicon). Meniscal root tears were repaired using No. 0 FiberWire or 0.9–1.3 mm FiberLink sutures (Arthrex) with transtibial fixation.

All patients followed a standardized postoperative rehabilitation protocol. In cases involving isolated ACLR or ACLR combined with meniscal resection, full weight‐bearing and unrestricted range of motion were permitted as tolerated. For patients who underwent meniscal repair, a hinged knee brace was worn for 6 weeks postoperatively. Flexion was restricted to 0–30° during the first 2 weeks, 0–60° during weeks 3 and 4, and 0–90° during weeks 5 and 6. Full weight‐bearing was allowed in all cases except those involving radial or root tears, for which weight‐bearing was restricted for 6 weeks postoperatively.

### STS laxity and extension deficit assessment

Instrumented anterior knee laxity and knee extension were measured both preoperatively and at six months following ACLR. Assessments were conducted at our outpatient clinic by experienced physical therapists specializing in sports medicine.

Anterior knee laxity was measured using the KT‐1000 arthrometer (MEDmetric). A standardized anterior load of 134 N (equivalent to 30 lb) was applied. Each knee was tested at least three times, and the median value was recorded. The postoperative STS difference in anterior tibial translation, comparing the operated knee to the uninjured contralateral side, was calculated and reported in millimeters. STS differences were categorized into three classes (≤ 2, 3–5, >5 mm) according to the IKDC form [11].

Passive range of motion was assessed with the patient in the supine position using a goniometer. The device was positioned over the lateral femoral epicondyle, with the proximal arm aligned to the lateral midline of the femur (referencing the greater trochanter) and the distal arm aligned to the lateral midline of the fibula (referencing the lateral malleolus) [7]. Extension deficit was defined as a knee angle difference of >5°, using anatomical zero as the reference [21].

### Statistical analyses

Data were summarized using descriptive statistics, including mean (standard deviation), median (25th–75th percentile), and frequency, as appropriate. Continuous outcomes were analyzed using repeated measures analysis of variance when data were normally distributed, with Bonferroni‐adjusted pairwise comparisons. If data were not normally distributed or were ordinal, the Friedman test was used with Wilcoxon signed‐rank tests and Bonferroni correction. Binary categorical variables were analyzed using Cochran's *Q* test with post hoc McNemar's tests and Bonferroni adjustment. A *p* < 0.05 was considered statistically significant. Analyses were performed using IBM SPSS Statistics, version 29.0 (IBM Corp.).

A post hoc power analysis using G*Power indicated that the study had >99% power (*β* < 0.01) to detect medium‐sized effects (*f* = 0.25) in repeated measures comparisons of mean STS laxity across the three femoral fixation groups at *α* = 0.05.

## RESULTS

A total of 864 patients were included, with 288 in each group: TightRope II RT, TightRope RT and EndoButton CL. The groups were comparable in age, sex and the presence of concomitant medial/lateral meniscal resection or cartilage injuries. However, time from injury to surgery was significantly shorter in the TightRope II RT group compared to EndoButton CL (5.6 vs. 8.1 months, *p* = 0.030) (Table 1).

**Table 1:** Patient demographics and surgical characteristics.

					Test between‐groups, Bonferroni‐adjusted *p* values		
	TightRope II RT	TightRope RT	EndoButton CL	*p* value	TRII vs. TR	TRII vs. EB	TR vs. EB
*n* patients	288	288	288				
Age, mean (y) ± SD	29.9 ± 9.5	29.8 ± 9.5	29.9 ± 9.5	n.s.			
Female	147 (51.0)	147 (51.0)	147 (51.0)	n.s.			
Time to surgery, median (m) [IQR]	5.6 (3.0−13.6)	6.7 (3.7−14.0)	8.1 (4.0−18.3)	0.036	n.s.	0.030	n.s.
Medial meniscus							
Injury	92 (31.9)	76 (26.4)	65 (22.6)	0.005	n.s.	0.006	n.s.
Resection	33 (11.5)	33 (11.5)	33 (11.5)	n.s.			
Suture	58 (20.1)	36 (12.5)	16 (5.6)	<0.001	0.045	<0.001	0.015
Lateral meniscus							
Injury	90 (31.3)	54 (18.8)	54 (18.8)	<0.001	<0.001	<0.001	n.s.
Resection	34 (11.8)	34 (11.8)	34 (11.8)	n.s.			
Suture	54 (18.8)	17 (5.9)	7 (2.4)	<0.001	<0.001	<0.001	n.s.
Cartilage injury	55 (19.1)	49 (17.0)	54 (18.8)	n.s.			

*Note*: Values are presented as number (%) unless otherwise specified. Missing values: Tegner, TRII (*n* = 5), TR (*n* = 18), EB (*n* = 0); Time to surgery, TRII (*n* = 12), TR (*n* = 5), EB (*n* = 4).

Abbreviations: EB, EndoButton CL; IQR, interquartile range; m, months; n.s., not significant; TR, TightRope RT; TRII, TightRope II RT; y, years.

### Meniscal and cartilage injury

While the number of meniscal resections was matched across groups, the prevalence and treatment of meniscal injuries varied. Medial meniscus suturing was most common in the TightRope II RT group (58/92, 63.0%), followed by TightRope RT (36/76, 47.7%) and EndoButton CL (16/65, 24.6%). Similarly, lateral meniscus suturing was more frequent in the TightRope II RT group (54/90, 60.0%) compared to TightRope RT (17/54, 31.5%) and EndoButton CL (7/54, 13.0%). Cartilage injuries ranged from 17.0% to 19.1% between the groups, with no statistically significant differences.

### Anterior knee laxity

**Figure 1:**
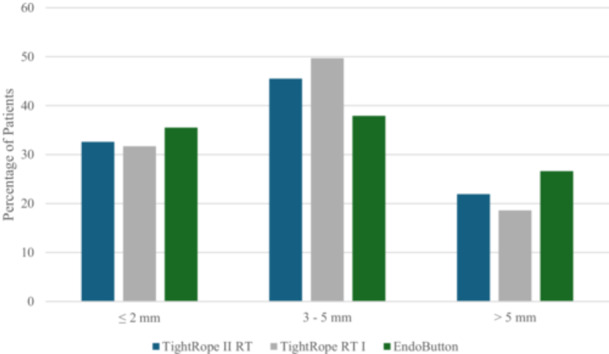
Preoperative side‐to‐side (STS) differences in anterior knee laxity measured by KT‐1000. Distribution of patients in each group based on STS difference at baseline (≤2, 3–5, >5 mm). No statistically significant differences were observed between groups.

**Figure 2:**
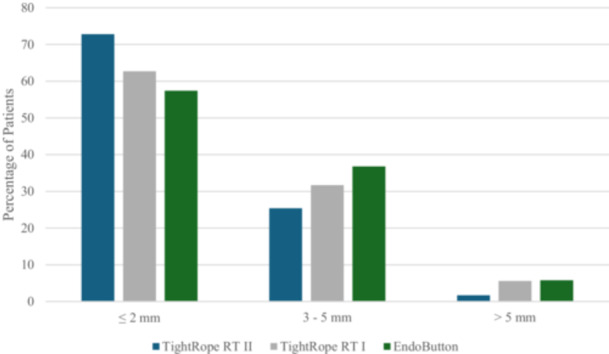
Side‐to‐side (STS) differences in anterior knee laxity at six‐month follow‐up measured by KT‐1000. STS category distribution (≤2, 3–5, >5 mm) by group. No statistically significant differences were observed between groups.

Anterior STS laxity measurements at baseline and 6 months are presented in Table 2. Mean STS differences did not differ significantly between groups. Moreover, there were no significant differences in the distribution of STS categories between the groups preoperatively or at 6 months postoperatively (Figures 1 and 2).

**Table 2:** Side‐to‐side (STS) differences measured by KT‐1000.

	TightRope II RT	TightRope RT	EndoButton CL	*p* value
**Preoperative**				
*n* patients	288	199	282	
STS, mean ± SD (mm)	3.8 ± 2.4	3.6 ± 2.4	3.8 ± 3.0	n.s.
**Postoperative (6 months)**				
*n* patients	287	142	258	
STS, mean ± SD (mm)	1.7 ± 1.6	1.8 ± 2.5	2.0 ± 2.3	n.s.

Abbreviations: EB, EndoButton CL; n.s., not significant; STS, side‐to‐side difference in anterior tibial translation.

### Extension deficit

Preoperative extension deficit was observed in 1.4% (4/287) of patients in the TightRope II RT group, 0.3% (1/280) in the TightRope RT group, and 0.7% (2/288) in the EndoButton CL group. At the 6‐month follow‐up, extension deficit was present in 6 patients: 0.4% (1/245) in the TightRope II RT group, 1.5% (3/206) in the TightRope RT group, and 0.8% (2/261) in the EndoButton CL group. No statistically significant differences were observed between groups at either time point.

## DISCUSSION

The main finding of this study was that TightRope II RT resulted in STS laxity and extension deficit proportions comparable to those of TightRope RT and EndoButton CL at 6 months postoperatively. This suggests that TightRope II RT is at least as effective as the other femoral fixation devices with respect to short‐term anterior knee laxity and early postoperative extension outcomes following ACLR.

The findings of this study are consistent with the growing body of literature showing no clinically important differences in anterior knee laxity between adjustable‐loop and fixed‐loop cortical suspension devices [9, 12, 19]. Ranjan et al. conducted a prospective randomized trial comparing the first‐generation TightRope RT with EndoButton CL and reported no significant differences in KT‐1000‐measured laxity at 6 months or 2 years postoperatively [19]. Similarly, Heng et al. found no difference in STS laxity at 6, 12, or 24 months when comparing an adjustable‐loop device (ToggleLoc) to EndoButton CL [12]. In a recent nationwide registry study, Elmholt et al. concluded that adjustable‐loop devices were noninferior to fixed‐loop buttons regarding anterior laxity and pivot shift and were also associated with lower mean STS differences 1 year after surgery [9].

Concerns have been raised regarding the potential for adjustable‐loop devices to lengthen under cyclic loading, possibly increasing postoperative laxity [13]. However, both biomechanical and clinical studies suggest this risk is minimal with modern designs [1, 14, 18, 22]. Onggo et al. concluded in a systematic review that elongation and fixation strength are comparable between adjustable‐ and fixed‐loop devices when proper surgical technique is used, specifically including retensioning of the adjustable loop after tibial fixation [18]. Similarly, Hyodo et al. observed only a minimal loop elongation of approximately 1 mm on serial magnetic resonance imaging over 1 year, with no association to increased laxity or inferior outcomes [14]. Smith et al. found no significant biomechanical differences between adjustable‐ and fixed‐loop devices and concluded that adjustable loops are comparable to fixed loops when retensioned [22]. Clinical findings by Boyle et al. also showed no differences in knee laxity or graft failure between fixation types [1]. Taken together with the present findings, which showed no significant differences in mean STS laxity or STS category distribution at 6 months, these results suggest that adjustable‐loop devices, including TightRope II RT, can maintain early postoperative anterior knee laxity comparable to fixed‐loop alternatives.

Increased postoperative STS laxity at 6 months (≥3 mm) has been associated with inferior long‐term outcomes, including a higher risk of revision or graft failure, persistent laxity, reduced Lysholm scores, and shortened athletic careers [5, 16]. Additionally, STS values >5 mm at 1 year have been linked to a more than fivefold increased risk of revision compared to values ≤2 mm [10]. Interestingly, although not statistically significant, the distribution data showed that TightRope II RT had the highest proportion of patients with an STS difference ≤2 mm and the lowest proportions in the 3–5 and >5 mm categories. However, this pattern may be influenced by the higher rate of meniscal repair in the TightRope II RT group, as meniscal preservation has previously been associated with improved postoperative knee laxity [5]. Further studies with longer follow‐up are needed to determine whether this translates into improved graft survivorship and patient‐reported outcomes.

Postoperative extension deficit was rare in all groups, with no significant differences observed between patients treated with TightRope II RT, TightRope RT, or EndoButton CL. This finding aligns with previous observations of comparable knee extension across different femoral fixation methods [12]. Although overtensioning of the graft has been recognized as a potential cause of extension deficit, the underlying cause of the few cases observed in this cohort remains unclear but could be due to factors such as graft impingement, arthrofibrosis, or suboptimal graft placement [21].

A major strength of this study is the large, well‐matched cohort comparing three widely used femoral fixation devices in ACLR. Matching was performed on clinically relevant variables, including age, sex, time to surgery, meniscal resection, and cartilage injury, which strengthens the internal validity of the findings [2, 4, 6]. Additionally, outcomes were assessed using standardized, objective measures of anterior knee laxity (KT‐1000) and knee extension at a uniform 6‐month follow‐up. All surgeries were performed at a single institution, ensuring consistency in surgical technique and postoperative rehabilitation protocols.

However, several limitations must be acknowledged. First, the retrospective design limits the ability to control for all potential confounding factors, and causality cannot be established. Second, the follow‐up period was limited to 6 months, precluding assessment of long‐term outcomes such as re‐rupture rates. Third, surgical technique–related variables, such as tunnel placement and graft tensioning, were not controlled for, as these data were not available. Fourth, postoperative KT‐1000 data were only available for approximately half of the patients in the TightRope RT group, which may have reduced the reliability and precision of between‐group comparisons. Fifth, although KT‐1000 and knee extension assessments were performed by experienced physical therapists, some degree of interobserver variability cannot be excluded. Additional limitations include possible differences in graft configurations, as patients receiving semitendinosus–gracilis grafts may experience greater laxity than those with semitendinosus‐only grafts [3]. There were also differences in the rate of meniscal repair among patients with meniscal injuries, despite matched numbers of meniscal resections across groups. Moreover, despite matching on time to surgery, a residual difference remained between TightRope II RT and EndoButton CL, likely reflecting the limitations of category‐based matching. Finally, because TightRope II RT is a newer implant, differences in the year of surgery between groups may have introduced confounding related to temporal trends or patient selection over time, although surgical technique and rehabilitation protocols were consistent throughout the study period.

## CONCLUSION

TightRope II RT provided short‐term outcomes comparable to TightRope RT and EndoButton CL in ACLR, with similar anterior STS laxity and knee extension deficit six months postoperatively. These findings support the use of TightRope II RT as a reliable femoral fixation method.

## AUTHOR CONTRIBUTIONS

All authors contributed to the conception and design of the study. Anders Stålman and Riccardo Cristiani extracted the data. Dzan Rizvanovic conducted the analyses and wrote the initial draft of the manuscript. Christoffer von Essen, Riccardo Cristiani and Anders Stålman critically revised the manuscript. All authors (Dzan Rizvanovic, Christoffer von Essen, Riccardo Cristiani, Anders Stålman) had full access to the data, participated in interpretation of the findings, and approved the final version. Anders Stålman is the study guarantor.

## CONFLICT OF INTEREST STATEMENT

The authors declare no conflicts of interest.

## ETHICS STATEMENT

The study was approved by the Swedish Ethical Review Authority (2024‐07166‐02).

## Data Availability

The data supporting the findings of this study are available from the corresponding author upon reasonable request and approval from Capio Artro Clinic, Stockholm, Sweden, and from the Swedish Ethical Review Authority.
